# Identifying Mitotic Kinesins as Potential Prognostic Biomarkers in Ovarian Cancer Using Bioinformatic Analyses

**DOI:** 10.3390/diagnostics12020470

**Published:** 2022-02-12

**Authors:** Hailun Liu, Chen Chen, Tanja Fehm, Zhongping Cheng, Hans Neubauer

**Affiliations:** 1 Department of Obstetrics and Gynecology, University Hospital and Medical Faculty of the Heinrich-Heine University Duesseldorf, Universitaetsstr, 1, 40225 Duesseldorf, Germany; hailun.liu@med.uni-duesseldorf.de (H.L.); chen.chen@med.uni-duesseldorf.de (C.C.); tanja.fehm@med.uni-duesseldorf.de (T.F.); 2 Department of Obstetrics and Gynecology, Shanghai Tenth People’s Hospital, Tongji University School of Medicine, Shanghai 200072, China; 3 Breast and Thyroid Center, The First People’s Hospital of Zunyi (The Third Affiliated Hospital of Zunyi Medical University), Zunyi 563000, China; 4 Institute of Gynecological Minimally Invasive Medicine, Tongji University School of Medicine, Shanghai 200072, China

**Keywords:** mitotic kinesins, ovarian cancer, prognostic biomarkers, therapeutic targets, bioinformatic analyses

## Abstract

Ovarian cancer (OC) is characterized by late-stage presentation, chemoresistance, and poor survival. Evaluating the prognosis of OC patients via effective biomarkers is essential to manage OC progression and to improve survival; however, it has been barely established. Here, we intend to identify differentially expressed genes (DEGs) as potential prognostic biomarkers of OC via bioinformatic analyses. Initially, a total of thirteen DEGs were extracted from different public databases as candidates. The expression of KIF20A, one of the DEGs, was correlated with a worse outcome of OC patients. The functional correlation of the DEGs with mitosis and the prognostic value of KIF20A imply a high correlation between mitotic kinesins (KIFs) and OC development. Finally, we found that KIF20A, together with the other nine mitotic KIFs (4A, 11, 14, 15, 18A, 18B, 23, C1, and2C) were upregulated and activated in OC tissues. Among the ten, seven overexpressed mitotic KIFs (11, 14, 18B, 20A, 23, and C1) were correlated with unfavorable clinical prognosis. Moreover, KIF20A and KIF23 overexpression was associated with worse prognosis in OC patients treated with platinum/taxol chemotherapy, while OCs overexpressing mitotic KIFs (11, 15, 18B, and C1) were resistant to MAPK pathway inhibitors. In conclusion, worse outcomes of OC patients were correlated with overexpression of several mitotic KIFs, which may serve both as prognostic biomarkers and therapeutic targets for OC.

## 1. Introduction

Ovarian cancer (OC), a molecularly heterogeneous disease, is a major cause of death among gynecological malignancies [1,2]. It is generally characterized by non-specific early clinical symptoms, advanced stage diagnosis, and poor survival. The current overall 5-year survival rate is close to 90% in early stages (I + II) but only 29% in late stages (III + IV) [3,4]. Standard therapy comprising tumor-debulking surgery and platinum/taxane chemotherapy has been used in OC treatment for decades, resulting in a significant increased survival. However, most OC patients relapse due to chemoresistance, causing treatment failure and more than 90% of deaths [5]. Late diagnosis, drug resistance, and high recurrence are still the major issues threatening to the prognosis of OC patients [6]. Therefore, exploring newly valuable prognostic biomarkers for promoting the survival rate of OC patients is urgently needed. Such an effective prognostic biomarker shall measure the association between the OC disease and clinical outcome either in the absence or presence of standard therapy to improve the OC treatment [7,8]. It is distinguished from a predictive biomarker, which identifies factors associated with the effect of intervention or exposure [7]. However, barely effective biomarkers have been established to measure the prognosis of OC patients.

Identifying a prognostic biomarker requires determining its potential relevance before validating its clinical utility and utilization [7,8]. Cancer bioinformatics is one of the several ways to detect biomarkers related to diagnoses, to monitor disease progression, and response to therapies [9]. The analysis of DEGs by RNA-sequencing (RNA-seq) is a common approach to obtain considerable insights into, e.g., resistance mechanisms [10].

KIFs are a large superfamily of microtubule-based motor proteins required for mitosis and intracellular transport [11]. Currently, there are sixteen KIFs (2A/B/C, 4A/B, 10, 11, 14, 15, 18A/B, 20A/B, 22, 23, and C1), which are categorized as mitotic KIFs due to their coordinating function in mitosis and cytokinesis, which represent critical phases in cell cycle required for cell growth and development [12]. A bench of data suggests that dysregulated cellular proliferation and cancer development are highly correlated [13,14]. Furthermore, increasing evidence has implied that chemoresistance and high recurrence in OC patients may be tightly associated with overexpressed KIFs [14]. For instance, KIF20A is mainly involved in cellular proliferation, migration, invasiveness, and angiogenesis and is significantly upregulated in different types of cancers [15,16,17]. Other extensively studied mitotic KIFs, such as KIF11 (Eg5/KSP) and KIFC1 (HSET), have attracted significant attention in searching for alternative mitotic drug targets to overcome chemoresistance [18]. Considering that the mitotic KIFs play a critical role in mitosis and that they are highly correlated with cancer development, we aimed to apply bioinformatic data analyses to uncover their potential values as prognostic biomarkers and therapeutic targets and to reveal new insights into OC treatment.

## 2. Materials and Methods

### 2.1. Identification of DEGs

Four public datasets were screened and interrogated for genes that are silenced in the normal tissues, including Human Protein Atlas (HPA) (19,651 genes in 43 tissues), the Genotype-Tissue Expression (GTEx) (46,711 genes in 53 tissues), the Illumina Body Map (49,311 genes in 16 tissues), and the RIKEN FANTOM5 (21,105 genes in 76 tissues). The silenced genes are defined as: <1NX (Normalized Expression) or <1 TPM (Transcripts Per Million). The up-regulated genes in OC tissues were investigated from the TCGA-OV dataset via the cBioPortal website. The inclusion criteria are (1) OC patients with complete transcriptional data, and (2) the definition of significantly up-regulated genes is the minimal expression value >10 RSEM (RNA-Seq by Expectation-Maximization).

### 2.2. Gene Annotations

Annotations of DEGs were obtained from the Ensembl Genome Browser [19] (http://www.ensembl.org/index.html) (accessed on 2 February 2022), which offers an integrated and reusable framework for generating, sorting, retrieving, and displaying genomic annotation data. The annotations of DEGs include Ensembl gene IDs, chromosome locations, gene types, transcripts number, and protein functions.

### 2.3. Expression Profiling Analysis for Mitotic KIFs

GEPIA (http://gepia2.cancer-pku.cn/index.html) (accessed on 2 February 2022) is a valuable and highly cited resource for gene expression analysis based on tumor and normal samples from the TCGA (http://tcgaprotal.org/index.html) (accessed on 2 February 2022) and GTEx (http://gtexprotal.org/home/index.html) (accessed on 2 February 2022) datasets [20]. We performed differential expression analysis of mitotic KIFs RNA sequences data of 426 OC and 88 normal ovarian samples using GEPIA.

UALCAN (http://ualcan.path.uab.edu/) (accessed on 2 February 2022) is a database for deep mining of TCGA data, which can be utilized to analyze gene transcription levels in clinicopathological subgroup [21]. In this study, we employed the analysis function of the UALCAN database to compare the stages and grades for the ten overexpressed mitotic KIFs in mRNA level.

The Human Protein Atlas (HPA) (https://www.proteinatlas.org) (accessed on 2 February 2022) is a website that contains immunohistochemistry-based expression data for nearly 20 highly common cancers [22]. In this study, a direct comparison of protein expression of mitotic KIFs between human normal ovarian and OC tissues was performed by immunohistochemistry images. Annotation parameters include an evaluation of: (1) staining intensity (not detected, weak, moderate, strong); (2) fraction of stained cells (rare, <25%, 25–75%, >75%); and (3) subcellular localization (nuclear and/or cytoplasmic membranous). Antibodies selected for each gene were kept identical for better comparison.

### 2.4. Survival Analysis

Kaplan–Meier Plotter (http://www.kmplot.com) (accessed on 2 February 2022), an online database for comprehensive prognosis analysis, was used to assess the prognostic significance of the mRNA expression levels of DEGs and mitotic KIFs genes. For analyzing the OC patients’ overall survival (OS), progression-free survival (PFS), and post-progression survival (PPS), all samples were stratified into low- or high-expression groups according to the 50% median expression level. Hazard ratios (HR), 95% confidence intervals (95% CI), and *p*-value were auto-calculated by the Kaplan–Meier Plotter.

### 2.5. Function Enrichment Analysis

Metascape (http://metascape.org) (accessed on 2 February 2022) has integrated more than 40 bioinformatic knowledge bases, which enables identification of enriched pathways [23]. Metascape enrichment analysis employed hypergeometric test and Benjamini–Hochberg methods to filter statistically significant ontology terms [24]. The DEGs and mitotic KIFs enrichments were analyzed using the Gene Ontology (GO) approach, including biological process (BP), cellular component (CC), and molecular function (MF) categories.

### 2.6. Pathway and Drug-Sensitivity Analysis

GSCALite (http://bioinfo.life.hust.edu.cn/web/GSCALite/) (accessed on 2 February 2022) is a web-based analysis platform for gene set cancer analysis [25]. The correlation between mitotic KIFs with pathway activity and drug sensitivity was assessed using the pathway activity module and drug-sensitivity module separately. The linear correlation between the expression of mitotic KIFs and the 265 small molecules from Genomics of Drug Sensitivity in Cancer (GDSC) was analyzed using the Spearman’s correlation coefficient. These analyses were performed using the TCGA-OV dataset.

### 2.7. Immune Infiltration and Genetic Alterations Analysis

TIMER2.0 (http://timer.cistrome.org) (accessed on 2 February 2022) is a bioinformatic tool to comprehensively investigate the molecular characterization of tumor-immune interactions. Levels of tumor-infiltrating immune subsets provide various analyses with the dataset of 10,897 tumors from 32 cancer types [26]. Overexpressed mitotic KIFs and their correlation with the abundance of immune cells was evaluated using Spearman’s correlation with TCGA-OV dataset (*n* = 303).

The cBioPortal (http://www.cbioportal.org) (accessed on 2 February 2022) is an open-access website resource for exploring, visualizing, and analyzing multidimensional cancer genomics data [27]. The TCGA-OV dataset (Firehose Legacy) in cBioPortal was used to analyze the genomic profiles of overexpressed mitotic KIFs in OC tissues. Genomic data types are comprised of somatic mutations, i.e., copy-number alterations (CNAs).

### 2.8. Statistics

For all the analyses done above, a *p*-value < 0.05 is considered statistically significant except for specifically mentioned.

## 3. Results

### 3.1. Detection of Prognostic Biomarkers

We initially aimed at a special class of DEGs that is silenced in normal ovarian tissues but upregulated in OC tissues with a potentially key role in developing or maintaining OC. These specific DEGs may have clinical value as prognostic biomarkers or therapeutic targets for OC treatment.

**Figure 1:**
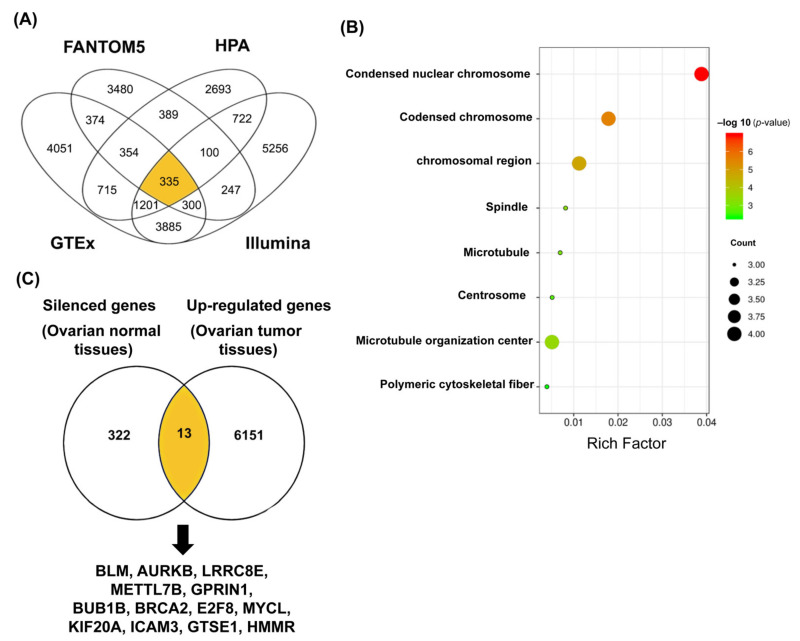
Identification and functional characterization of DEGs in OC. (**A**) Four datasets (FANTOM5, HPA, GTEx, and Illumina Body Map) were used to identify silenced genes in normal ovarian tissues, and the 335 overlap genes were marked by yellow in the middle; (**B**) 335 silenced genes from normal ovarian tissues (left) and 6164 up-regulated genes in OC tissues from TCGA-OV dataset (right); the 13 overlap genes were marked by yellow in the middle. These 13 common genes (DEGs) are silenced in normal ovarian tissues but up-regulated in OC tissues; (**C**) scatter plot of enriched GO pathway statistics. Rich factor is the ratio of the DEGs number to the total gene numbers in a certain pathway. The color and size of the dots represent the range of *p*-value (hypergeometric test and Benjamini–Hochberg methods) and the number of DEGs mapped to the indicated pathways. Top 10 enriched pathways are showed in the figure.

To eliminate the system bias, we screened all silenced genes in normal ovarian tissues from four datasets: 6509 genes in HPA; 11,215 genes in GTEx; 12,046 genes in Illumina Body Map; and 5579 genes in RIKEN FANTOM5 (Figure 1A) based on the definition of silenced genes (<1NX (Normalized Expression) or <1 TPM (Transcripts Per Million)). Among these, a total of 335 genes are common across the four datasets. In parallel, we obtained 6146 significantly up-regulated genes in OC tissues across 600 patients from the TCGA-OV dataset (Figure 1B). The clinical and pathological characteristics of OC patients from the TCGA-OV dataset are summarized in Table 1. Thirteen common genes between the silenced genes from normal ovarian tissues and the up-regulated genes from OC tissues were observed, including HMMR, GTSE1, ICAM3, KIF20A, MYCL, E2F8, BRCA2, BUB1B, GPRIN1, METTL7B, LRRC8E, AURKB, and BLM, and characterized as DEGs (Figure 1B). To get a better understanding of these DEGs, gene annotations were obtained via the Ensembl genome browser. The Ensembl gene IDs, chromosome locations, transcripts numbers, gene types, and protein functions are shown in Table 2.

Then, gene ontology (GO) enrichment analysis was performed to understand biological processes associated with DEGs in OC cells. The pathway enrichment analysis in Metascape showed that these DEGs are mainly involved in cellular proliferation, such as regulation of cell cycle process (BLM, BRCA2, BUB1B, HMMR, AURKB, KIF20A, GTSE1, and E2F8), cytokinesis (BRCA2, AURKB, KIF20A, and E2F8), and mitotic cell-cycle checkpoints (BLM, BUB1B, AURKB, GTSE1, and E2F8) (Figure 1C).

### 3.2. Correlation of Overexpressed of KIF20A/BRCA2/BUB1B with Poor Prognosis in OC

**Figure 2:**
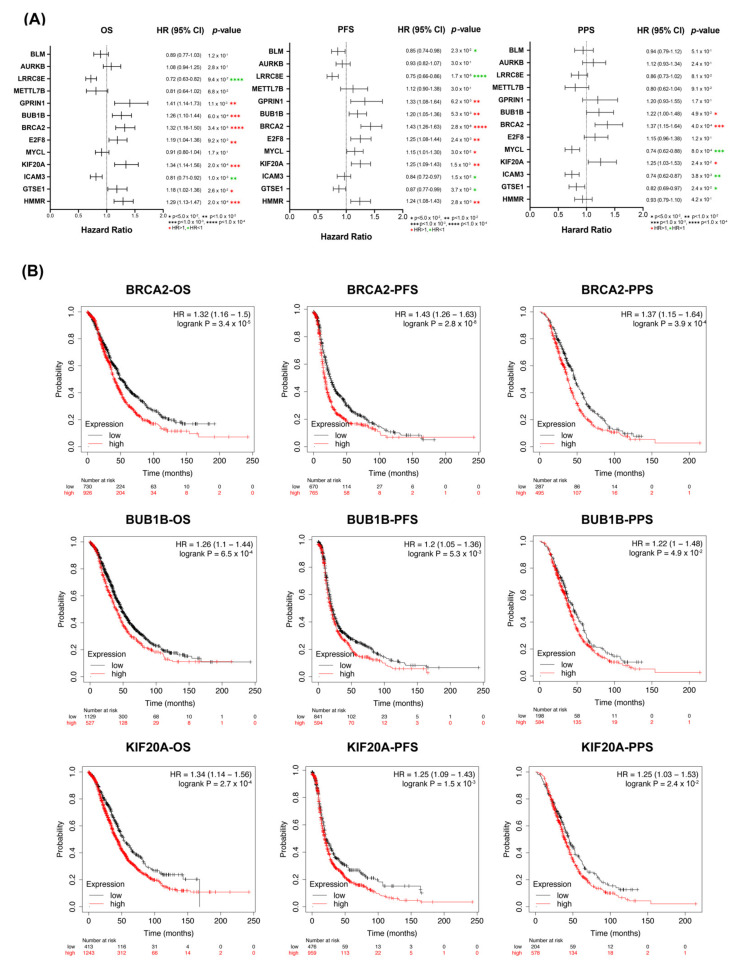
Prognostic values of 13 DEGs in OC. (**A**) Forest plots of 13 DEGs with survival analyses regarding OS, PFS, and PPS using TCGA-OV dataset; (**B**) survival analyses of BRCA2, BUB1B, and KIF20A regarding OS, PFS, and PPS using TCGA-OV dataset. Red, high-expression group; Black, low-expression group. *p*-value is log-ranked. Auto-selected best cutoff was used.

We then used the Kaplan–Meier Plotter to analyze the prognostic values of the 13 DEGs in OC cells. As shown in Figure 2A and in the Supplementary Figure S1, the up-regulated expression levels of each BUB1B, BRCA2, and KIF20A was correlated with worse overall survival (OS), post-free survival (PFS), and post-progression survival (PPS). Overexpressed GPRIN1, E2F8, and HMMR, respectively, were correlated with worse OS and PFS but not PPS. Overexpressed GTSE1 was significantly associated with an unfavorable PFS. No significance was observed between clinical outcomes with high expression levels of METTL7B and AURKB. OCs overexpressing LRRC8E, MYCL, and ICAM3 had better clinical outcomes. Besides, high expression of BLM was correlated with a better PFS.

In summary, measuring the expression levels of KIF20A, BRCA2, and BUB1B may be of comprehensive prognostic value for OC patients’ survival (Figure 2B). In line with previous studies, BRCA2 and BUB1B are up-regulated genes in OC, and their overexpression may correlate with OC development [28,29,30], and KIF20A (also known as MKLP2) may be an indicator to predict unfavorable outcomes in ovary clear-cell carcinoma [31]. Compared to BRCA2 and BUB1B, the functional and prognostic value of KIF20A is less well investigated in OC.

### 3.3. Overexpression of Further Nine Mitotic KIFs as Well as KIF20A in OC

The overexpression of KIF20A and its correlation with worse clinical outcome indicate that it may serve as a prognostic biomarker of OC. Meanwhile, the prognostic values of other mitotic KIFs are barely characterized in OC. This raises the question of whether their expression levels are upregulated and correlated with worse clinical outcomes as well.

**Figure 3:**
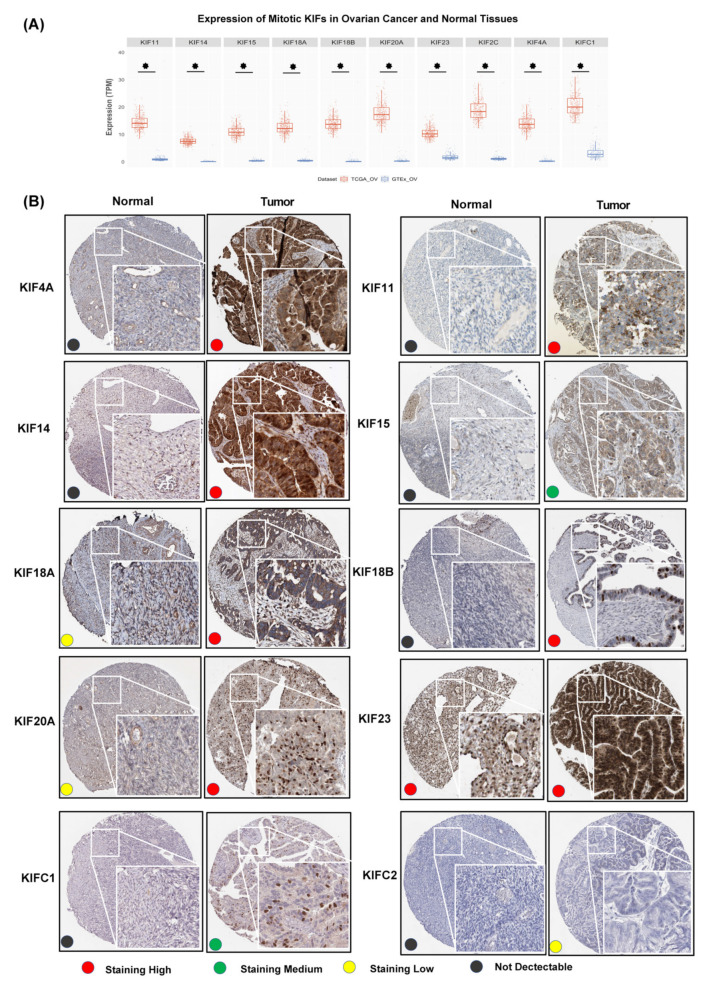
Expression profiles of mitotic kinesin superfamily in OC. (**A**) mRNA levels of ten overexpressed mitotic KIFs (4A, 11, 14, 15, 18A, 18B, 20A, 23, C1, and 2C) in ovarian cancer and normal ovarian tissues from TCGA-OV (*n* = 426) and GTEx-OV (*n* = 88) dataset, respectively. TPM, transcripts per million; * *p* < 0.05; (**B**) Immunohistochemistry images of ten overexpressed mitotic KIFs in ovarian cancer and normal ovarian tissues from Human Protein Atlas. Red, green, yellow, and black dots present high, medium, low staining, and not detectable, respectively. KIFs (4A, 11, 14, 15, 18B, C1, and 2C) protein were not expressed in normal ovarian tissues; KIF18A and KIF20A have low expression in cytoplasmic/membranous. KIF23 has relative high expression in cytoplasmic/membranous. KIFs (4A, 11, 14, 18A, 18B, 20A, and 23) have high expression in OC tissues, and KIF15 and KIFC1 have medium expression, whereas KIF2C expression level is relatively low in OC tissues.

We further compared the expression levels of mitotic KIFs between OC and normal ovarian tissues to identify which mitotic KIFs is up-regulated in OC tissues in addition to KIF20A. Clustering analysis resulted in ten out of sixteen mitotic KIFs (4A, 11, 14, 15, 18A, 18B, 20A, 23, C1, and 2C) were significantly overexpressed in OC tissues (Figure 3A and Supplementary Figure S2). This was further supported by immunocytochemistry data (Figure 3B): the ten identified mitotic KIFs are barely detected in normal ovarian tissues. KIFs 18A and 20A exhibited weak cytoplasmic and membranous expression levels, while KIF23 was strongly present in normal ovarian stromal cells. In contrast, nine of ten mitotic KIFs exhibit increased IHC signals in OC tissues. KIFs 4A, 11, 15, and 18A primarily localized to the cytoplasm and membrane, while KIFs 14, 18B, 20A, 23, and C1 localized to the nucleus. The remaining KIF2C showed a high mRNA level (Figure 3A) but low protein level (Figure 3B).

### 3.4. No Correlation of Overexpressed Mitotic KIFs with Tumor-Infiltrating Lymphocytes

Given that the presence of tumor-infiltrating lymphocytes (TILs) is related to prognosis, identification of the tumor immune microenvironment (TIME) in OC was thought to be meaningful [32]. The correlation between the overexpression of mitotic KIFs and the presence of TILs was systematically analyzed with TIMER2.0 (Supplementary Figure S3). The strongest correlation was observed for the expression of KIF4A and the presence of neutrophil cells, but the correlation coefficient was low (cor = 0.299, *p* = 1.54 × 10^−6^). In conclusion, none of the overexpressed mitotic KIFs was significantly correlated with any types of TILs, including CD4+ T cells, CD8+ T cells, B cells, neutrophils, macrophages, and myeloid dendritic cells.

### 3.5. Correlation of Overexpressed Mitotic KIFs with High Cellular Proliferation

Specific mutations or genetic variations enable tumor initiation or progression and influence the effectiveness of anticancer therapies [33]. Since the genetic background of a cells affects its gene expression, we asked if the abnormal activation of mitotic KIFs in OC is a result of the genetic events, such as copy number alterations and mutations.

**Figure 4:**
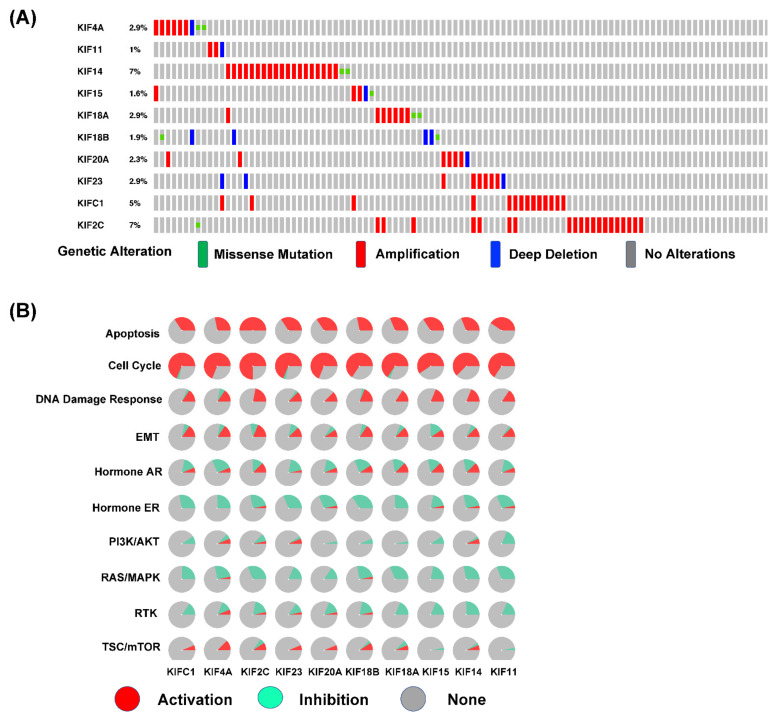
Genetic alterations and cancer-related pathways of ten overexpressed mitotic KIFs in OC. (**A**) Genetic alterations of ten overexpressed mitotic KIFs in OC (cBioPortal). Genetic mutation events include missense mutation, amplification, and deep deletion. KIF14 and KIF2C rank the relatively highest two genes of genetic alterations, and their mutation rates are both 7%; (**B**) the roles of ten overexpressed mitotic KIFs in the famous cancer-related pathways (GSCALite). The red, turquoise, and grey parts present activation, inhibition, and none, respectively.

According to the TCGA-OV dataset, copy number alterations (amplification and deep deletion) and mutations of each overexpressed mitotic KIFs rarely occur in OC (Figure 4A). Among 311 OC samples, the highest alteration rate of 7% was observed for both KIF14 and KIF2C. The highest deep deletion rate (a deep loss/homozygous deletion, log2CopyNumber <−1) was related to KIF18B, which was 1.28%. We also investigated the mutation profiles of the ten overexpressed mitotic KIFs in OC, and only two mutations each were found within KIF4A, KIF14, or KIF18A/B and one mutation each within KIF15 or KIF2C. No mutation was observed within KIF11, KIF20A, KIF23, and KIFC1 (Figure 4A). Taken together, these results imply that copy number alterations and mutations may not represent the main reasons for abnormal activation of mitotic KIFs in OC.

This raises the question of which cellular processes are correlated with the overexpressed mitotic KIFs. Our GO enrichments showed that the mitotic KIFs were mainly involved in cellular proliferation, including cell and nuclear division, mitotic nuclear division pathways, microtubule-based movement, and cytoskeleton organization (Supplementary Figure S3A–C, Table 3). We further explored the roles of the overexpressed mitotic KIFs in cancer-associated pathways, such as apoptosis, cell cycle, DNA damage response, epithelial-mesenchymal transition (EMT), hormone AR, hormone ER, PI3K/AKT, RAS/MAPK, and RTK pathway. Consistent with data from the GO enrichment analysis, the cell-cycle progression was highly associated with all mitotic KIFs in OC cells (Figure 4B). In addition, overexpressed mitotic KIFs play an essential role in apoptosis, DNA damage response, and EMT pathway as well. These results collectively suggest that overexpressed mitotic KIFs lead to activated mitosis of OC cells.

### 3.6. Expression Profile of Overexpressed Mitotic KIFs in Different OC Stages and Grades

**Figure 5:**
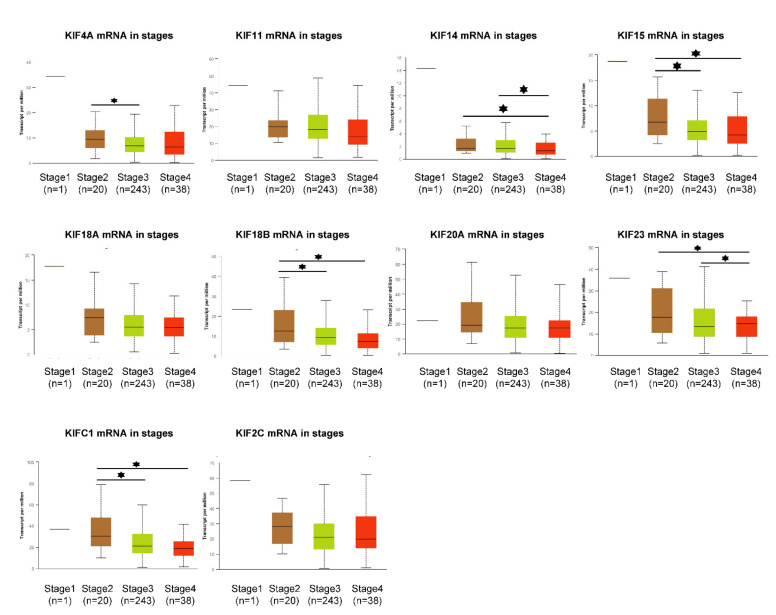
Transcription profiles of ten overexpressed mitotic KIFs in OC clinicopathological subgroup analysis (UALCAN). Sample numbers of each stage: stage 1 (*n* = 1); stage 2 (*n* = 20); stage 3 (*n* = 243); and stage 4 (*n* = 38). * *p* < 0.05.

To further dissect the expression levels of the identified ten overexpressed mitotic KIFs in different OC stages and grades, we performed a multivariate clinicopathological subgroup analysis by using the UALCAN database. The detailed results are depicted in Figure 5 and in Supplementary Figure S5. Intriguingly, the expression levels of almost all overexpressed mitotic KIFs declined from stage 2 to stage 4 although for KIFs 11, 20A, 18A, and 2C, this trend was not significant (Figure 5). As for clinical grades, the expression levels of the ten mitotic KIFs increased moderately or did not change significantly from grade 2 to grade 4. Only the expression of KIF20A and KIF23 increased significantly from grade 2 to grade 3 (Supplementary Figure S5). Note that the sample number (*n* = 1) for stage1, grade 1, and grade 4 is only 1, and a possible reason may be the late-stage presentation and poorly classified differentiation of OC diagnosis.

### 3.7. Overexpression of Survival-Related Mitotic KIFs Indicates Worse Prognoses in Early-Stage and Low-Grade OC Patients

**Figure 6:**
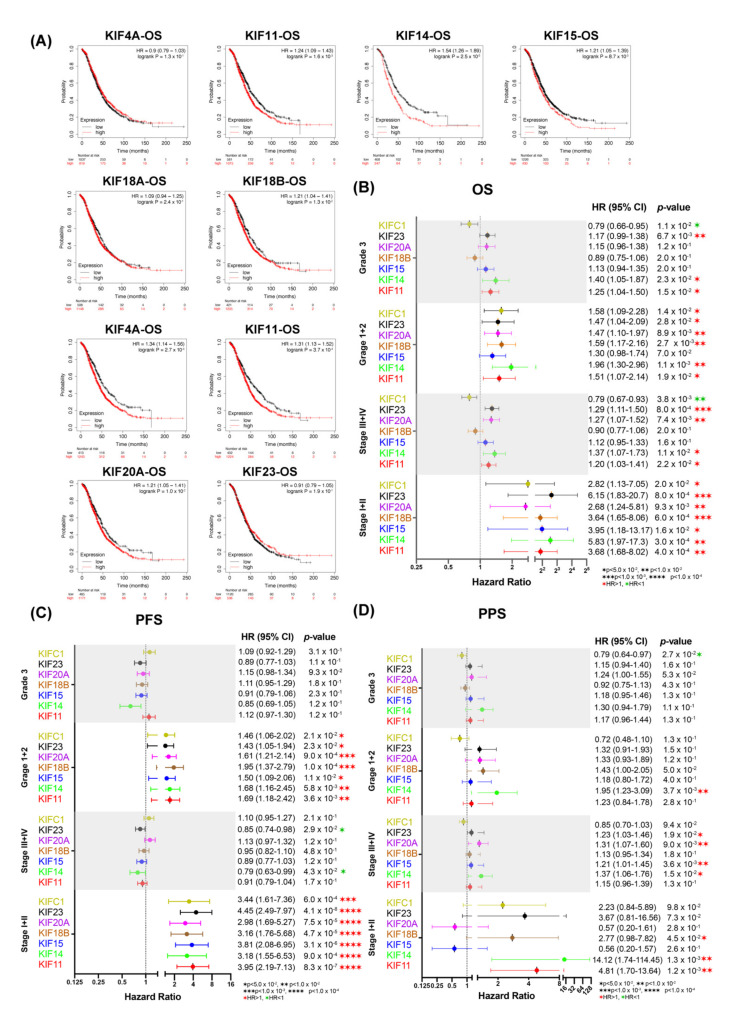
Prognostic values of ten overexpressed mitotic KIFs in OC. (**A**) Survival analyses of ten overexpressed mitotic KIFs regarding OS using TCGA-OV dataset. Red, high-expression group; Black, low-expression group. *p*-Value is log-ranked. Auto-selected best cutoff is used. Forrest plots of relationship between prognosis (**B**) OS, (**C**) PFS, (**D**) PPS, and ten overexpressed mitotic KIFs mRNA expression in patients with different OC clinicopathological features, including grades and stages.

The prognostic values of all overexpressed mitotic KIFs in OC were explored by Kaplan–Meier Plotter. Except for KIF4A, KIF18A, and KIFC2, the remaining seven overexpressed mitotic KIFs (11, 14, 15, 18B, 20A, 23, and C1) were highly correlated with worse OS. They were also mostly associated with worse PFS and PPS (except for KIF15, KIF18B, and KIFC1 in PPS) (Figure 6A and Supplementary Figure S6). This suggests that they may be potential prognostic biomarkers for OC and warrants further investigation.

The observation of decreased expression levels of mitotic KIFs with increasing OC stages may point towards an association of these overexpressed mitotic KIFs with patient survival within different OC stages. Our analysis showed that, accompanied with higher expression levels of mitotic KIFs in early-stage (I + II) OC, all seven overexpressed mitotic KIFs were associated with a worse OS and PFS (Figure 6B,C). This indicates that these overexpressed mitotic KIFs may serve as negative prognostic indicators for early-stage OC patients. Similar results were obtained for the overexpressed KIFs 14, 20A, and 23, which were correlated with shorter OS and PPS in late-stage OC (III + IV) (Figure 6B,C). Taking together, KIFs 14, 20A, and 23 could be used as indicators of poor prognosis for all OC stages, whereas KIFs (11, 15, 18B, and C1) may be specific biomarkers indicating poor prognostic at early-stage OC (Figure 6B–D).

In low-grade OC (grades 1 + 2/well and moderated differentiated), except for overexpressed KIF15, which was associated with worse PFS but not with worse OS, the other six overexpressed mitotic KIFs were associated with both worse OS and PFS (Figure 6B,C), while in high-grade (grade 3/poorly differentiated) OC, overexpressed KIF11 and KIF14 were also linked to worse OS (Figure 6B), and the remaining five mitotic KIFs had no significant prognostic value (Figure 6B–D).

### 3.8. Overexpression of Survival-Related Mitotic KIFs May Related to Chemoresistance

**Figure 7:**
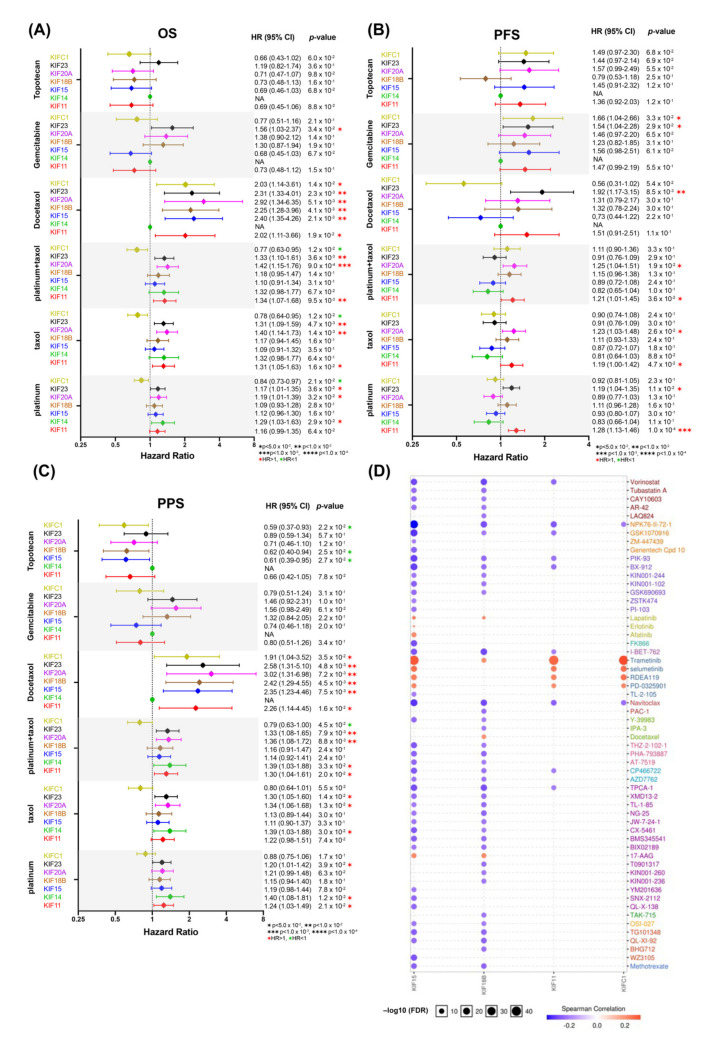
Prognostic values of ten overexpressed mitotic KIFs in OC chemotherapies. Forest plots of relationship between prognosis (**A**) OS, (**B**) PFS, (**C**) PPS, and ten overexpressed mitotic KIFs mRNA expression in patients with different OC chemotherapies using TCGA-OV dataset. (**D**) Drug resistance analyses of overexpressed mitotic KIFs. The expression of each gene was performed by Spearman correlation analysis with the small molecule/drug sensitivity (IC50). The positive correlation means that the gene high expression is resistant to the drug and vice versa.

A paclitaxel-platinum combination for OC patients treatment is used as first-line chemotherapy since decades, and other drugs, such as docetaxel, gemcitabine, and topotecan, are applied as second line [34]. Chemotherapy resistance is one of the major challenges for clinical outcomes [35]. Therefore, we evaluated the predictive roles of overexpressed mitotic KIFs in OC patients treated with platinum, taxol (also known as paclitaxel), docetaxel, gemcitabine, and topotecan by using the Kaplan–Meier Plotter database (Figure 7A–C). Overexpressed mitotic KIFs 11, 20A, and 23 showed poor association with OS in patients treated with either single-agent taxol or platinum/taxol combination or with single-agent platinum (Figure 7A–C). In the docetaxel-treated group, OC overexpressing all mitotic KIFs (except for KIF14) had poor OS and PPS survival outcomes (Figure 7A,C). Additionally, overexpressed KIF23 was correlated with shorter OS and PFS in gemcitabine-treated patients (Figure 7A,B). No significant predictive value exists for topotecan treatment (Figure 7A–C).

Base on the above results that several overexpressed mitotic KIFs may be of predictive value for the response to chemotherapy agents, we further evaluated their association with response to other drugs using the Genomics of Drug Sensitivity in Cancer (GDSC) database. The results revealed that OC cells with low expression of KIF15 and KIF18B were sensitive to most drugs or small molecules, but cells overexpressing KIFs 11, 15, 18B, and C1 were resistant to mitogen-activated protein kinase (MAPK kinase) inhibitors, such as Trametinib, Selumetinib, and RDEA119 (Figure 7D).

## 4. Discussion

Here, we provide data that overexpression of several mitotic KIFs is correlated with worse outcomes of OC patients. These mitotic KIFs may function as prognostic biomarkers and therapeutic targets for OC.

### 4.1. A New Insight Connects Mitotic KIFs with OC

The GO enrichment and the prognostic value analyses of 13 identified DEGs imply that mitosis and OC are correlated to quite a high level (Figure 1 and Figure 2). Overexpression of KIF20A was significantly correlated with poor oncologic outcomes and tumor progression in OC patients, which is consistent with previously reported studies [31,36]. Moreover, overexpression of KIF20A was correlated with various human cancers, such as gastric cancer, lung cancer, and breast cancer [37,38,39], implying a cancer-related function. The clinical significance of KIF20A in OC is reminiscent of other members of mitotic KIFs, whose expression profiles and potential prognostic values were sporadically identified in OC, and most of the studies only focused on a single kinesin member [40,41,42,43].

These data promoted us to connect all mitotic KIFs with OC and to comprehensively explore their prognostic role in this disease by comparing the expression levels of the mitotic KIFs between the normal ovarian tissues (GTEx) and OC tissues (TCGA-OV) using clustering analysis (Figure 3). Note that several mitotic KIFs are silenced in GTEx, such as KIF20A, which could be selected as one of the DEGs (Figure 1). One reason for this is that we applied a strict definition that candidate genes must be silenced in all four datasets (HPA, RIKEN FANTOM5, GTEx, and Illumina Body Map) to ensure that the results are reliable and reproducible.

We are perfectly aware that our results were purely performed in silico. However, they may be hypothesis generating and foster further investigations, e.g., to include protein expression since the analysis on transcription level cannot reflect global changes but only some aspects of the function of mitotic KIFs in OC. Additionally, future work may be extended to different histological OC subtypes, which has not been executed herein.

### 4.2. Hypothesis I: Survival-Related Mitotic KIFs Are the Potential Prognostic Biomarkers for the OC

Functionally, kinesins are divided into proteins with either mitotic or non-mitotic functions, which may be assembled/function in the mitotic spindle and intracellular transporting [44,45], respectively. Previous studies have revealed that overexpression of tumor-related mitotic KIFs correlates with worse outcomes of breast cancer patients and that they can be potential prognostic biomarkers [46,47]. Our results showed that ten out of sixteen mitotic KIFs were up-regulated on mRNA and protein levels (except for KIF2C) in OC tissues compared to normal tissues. Furthermore, the clinicopathological subgroup analysis resulted in ten mitotic KIFs expressed at high mRNA levels in OC stages II–IV and grades 2–3. With the limitation of the sample size of stage I (*n* = 1) and grade 1 (*n* = 1), the mRNA levels of these ten mitotic KIFs presents a converse result, which shows a high level at stage I and low level in grade 1. This raises the question of when these mitotic KIFs are upregulated during the progression of OC. Whether the overexpression starts at stage I/grade 1 or at stage II/grade 2 requires further exploration. The large sample size for stage I and grade 1 of OC patients may provide more information to answer this question.

The survival analyses revealed that seven out of ten overexpressed mitotic KIFs (11, 14, 15, 18B, 20A, 23, and C1) were correlated with worse OS and PFS, indicating that they could be potentially efficient prognostic biomarkers. This provides meaningful clues to study the role of these KIFs in tumorigenesis and progression of OC. More specifically, in early-stage (I + II) OC patients, both OS and PFS for patients with OCs overexpressing all seven mitotic KIFs were significantly worse than for patients with low expression levels. While at the late stage (III + IV), overexpressed KIFs (14, 20A, and 23) were correlated with shorter OS and PPS. In low-grade (1 + 2) OCs, the OS and PFS of patients with all six overexpressed KIFs (except for KIF15) were significantly worse than that of patients with low KIFs expression. High-grade (grade 3) OC patients overexpressing KIF14 and KIF11 exhibited shorter OS. These data suggest that KIFs 11, 15, 18B, and C1 are the potential prognostic indicators specifically for early-stage OC patients and that the remaining three KIFs 14, 20A, and 23 are potential prognostic biomarkers for all stages. Except for KIF15, these mitotic KIFs are the potential prognostic biomarkers for both early-stage and low-grade OC patients. KIF14 and KIF11 are potential prognostic biomarkers for high-grade OC patients. In summary, we proposed that these survival-related mitotic KIFs could guide clinical prognosis estimation especially in early-stage and low-grade OC patients.

### 4.3. Hypothesis II: Several Mitotic KIFs May Be Promising Therapeutic Targets for the OC Treatment

Normally, most patients suffering from low-grade and early-stage OC are closely observed following surgery or are treated with platinum- and taxol-based chemotherapy for 3–6 cycles, yielding response rates of over 80% [48,49]. Clinically, based on the length of the disease-free period, OCs are categorized into platinum-sensitive, platinum-resistant, and platinum-refractory cases. The last two groups of patients are usually treated with other agents, such as docetaxel, gemcitabine, topotecan, and hormonal therapies in the second line [50]. However, most of OC patients treated in this way will eventually relapse and will show chemoresistance at different levels with unknown mechanisms [51,52]. Our survival analysis indicated that overexpression of KIFs 11, 14, 20A, and 23 were associated with platinum/taxol drug resistance, affecting the patients’ prognosis, especially OS and PPS. In docetaxel-treated patients, overexpressed KIFs 11, 15, 20A, 23, and C1 may contribute to docetaxel chemoresistance. Overexpressed KIF23 and KIFC1 were also related to gemcitabine chemoresistance. Considering that binding targets of both mitotic KIFs and chemotherapeutic drugs are microtubules (except for gemcitabine, which targets DNA), chemoresistance may result from the competitive binding between overexpressed mitotic KIFs and these anti-cancer drugs to microtubule. Therefore, these mitotic KIFs not only could be used to identify patients who may or may not benefit from particularly targeted therapies. They may also be potential targets of specific drugs design, which requires further investigation. Consistently, the mitotic KIFs have obtained increasing attention in cancer research because of their essential roles during the cell cycle [53,54]. Data indicate that inhibiting a single pathway may limit its efficacy and may narrow the therapeutic indices, leading to resistance to the initial therapy [55]. Since complexes signaling networks mandate the necessity of drug combinations, the optimal efficacy of kinesin inhibitors as a part of a multidrug combination with traditional chemotherapy regimens has been studied in numerous clinical trials [56,57].

Given the high relapse rate and poor prognosis of OC, interests in the development of new treatment approaches will never stop. Several targeted molecular and biologic therapies, such as antiangiogenic agents, poly (ADP-ribose) polymerase inhibitors, signaling pathway inhibitors, and immunotherapies, have been widely researched [58,59,60]. The MAPK (mitogen-activated protein) pathway is involved in a variety of biological functions, including cell proliferation, mitosis, apoptosis, migration, and autophagy [61]. In recent years, there have been plenty of studies indicating that some mitotic KIFs and kinases involved in the MAPK pathway can cooperate to orchestrate several physiological processes [62,63]. Our drug-sensitivity analysis revealed that overexpressed KIFs (11, 15, 18B, and C1) influence the resistance against several MAPK inhibitors. Importantly, MAPK activation has also been associated with resistance to platinum-based chemotherapies in OC [64], which means that even though overexpressed KIFs (11, 15, 18B, and C1) did not directly influence worse outcomes in platinum-treated patients, mitotic KIFs may be potentially involved with MAPK activation. This may give a new perspective into the therapeutic value of mitotic KIFs. Therefore, further research should emphasize the inhibition of multiple mitotic KIFs and the MAPK signaling pathway in OC. These results display that overexpressed mitotic KIFs are involved in multiple chemotherapy resistance, highlighting their potentially important roles in chemotherapy.

### 4.4. Hypothesis III: Inherent Resistance of OC through Reduced Immonosurveillance and Subpopulations of Drug-Resistant OC

Most OC patients with recurrence after surgery and first-line chemotherapy are also resistant to second-line chemotherapy, which is a major clinical issue [65,66,67]. Growing evidence indicates that the nature of chemoresistance in OC is furthered by the existing subpopulations of drug-resistant OC cells [65,66,68]. Our data found that overexpression of several mitotic KIFs manifests a correlation with chemoresistance against drugs currently used in first- and second-line, thereby creating a functional connection between overexpressed mitotic KIFs and drug-resistant OC cells.

These drug-resistant OC cells may be derived from ovarian cancer stem cells (OCSCs), which can undergo genetic or epigenetic changes to generate microenvironmental immunosuppression and intertumoral heterogeneity (ITH) by dynamically interacting with the immunosurveillance and tumor microenvironment [68]. Most OC patients with tumor-infiltrating lymphocytes (TILs) are supposed to profit from a better outcome [68]; e.g., it was reported that OC patients whose tumors have intratumoral TILs experienced longer OS and PFS than patients whose tumors lacked TILs [69]. However, our immune infiltration analysis indicated that overexpression of mitotic KIFs was rarely correlated with almost all types of immune cells, including CD4+T cells, CD8+T cells, B cells, and macrophages, implying that OC cells with high expression levels of mitotic KIFs may generate a favorable immunosuppressive microenvironment to escape the immunosurveillance. The potential function of the overexpressed mitotic KIFs in evasion of the immunosurveillance requires more investigation in the future.

Taken together, both reduced immunosurveillance and drug-resistant OC cells were correlated with overexpressed mitotic KIFs in OC, which implies a worse outcome. This raises the possibility that the inherent chemoresistance of OC may result from a combination of reduced immunosurveillance and the OCSCs-derived, drug-resistant OC cells. Exploring the specific roles of overexpressed mitotic KIFs in maintaining and developing OCSCs may improve OC treatment by decreasing chemoresistance or increasing the anti-tumor immunity.

## 5. Conclusions

Although limited, our bioinformatic analyses provide hints that several overexpressed mitotic KIFs are correlated with worse outcomes of OC patients. They could therefore present new prognostic biomarkers and therapeutic targets for OC. As the importance of mitotic KIFs in tumor development and resistance formation has become evident, more exploration should be done.

## Figures and Tables

**Table 1:** Summary of clinical and pathological characteristics of 584 serous ovarian cancer patients from TCGA-OV dataset.

Characteristics	All Patients (*N*)	All Patients (%)
Age at diagnosis median, years		
<58	263	45.1
≥58	321	54.9
FIGO stage		
Early (I–II)	46	7.9
Late (III–IV)	535	91.6
NA	3	0.5
Histologic grade		
Low (G1–G2)	77	13.2
High (G3)	505	86.5
NA	2	0.30
OS status		
Living	227	38.9
Deceased	351	60.1
NA	6	1.0
OS median, months		
<32	285	48.8
≥32	296	50.7
NA	3	0.5
PFS median, months		
<14	239	41.0
≥14	259	44.3
NA	86	14.7

Abbreviation: TCGA, The Cancer Genome Atlas; FIGO, International Federation of Gynecology and Obstetrics; NA, not available; OS, overall survival; PFS, progression-free survival.

**Table 2:** Functional characterization of the 13 DEGs.

Gene Symbol Description	Ensemble ID	Chromosome	Transcripts Number	Gene Type	Protein Function
BLM	ENSG00000197299	15	11	Protein coding	DNA replication/repair, genome integrity
AURKB	ENSG00000178999	17	14	Protein coding	Cell-cycle regulation
LRRC8E	ENSG00000171017	19	6	Protein coding	Anion/aspartate transmembrane transport
METTL7B	ENSG00000170439	12	2	Protein coding	Methyltransferase activity
GPRIN1	ENSG00000169258	5	1	Protein coding	Neurite outgrowth, phosphoprotein binding
BUB1B	ENSG00000156970	15	11	Protein coding	Mitosis progression, ATP binding
BRCA2	ENSG00000139618	13	11	Protein coding	Double-strand break repair/homologous recombination
E2F8	ENSG00000129173	11	5	Protein coding	DNA binding transcription factor activity
MYCL	ENSG00000116990	1	4	Protein coding	DNA binding, protein dimerization activity
KIF20A	ENSG00000112984	5	7	Protein coding	Microtubule binding, ATPase activity
ICAM3	ENSG00000076663	19	10	Protein coding	Integrin and signaling receptor binding
GTSE1	ENSG00000075218	22	4	Protein coding	P53-induced cell-cycle arrest, Microtubule binding
HMMR	ENSG00000072571	5	8	Protein coding	Cell motility, cellular transformation, metastasis formation

**Table 3:** A summary of the seven overexpressed mitotic KIFs in cell division.

Mitotic KIFs	Kinesins Family	Localization	Cell Cycle Stage	Main Function
KIF11	Kinesin-5	Spindle/pole	Prophase, Metaphase	Bipolar spindle formation, separation of duplicated centrosomes
KIF14 (CMKRP)	Kinesin-3	Spindle/midbody	Telophase, Cytokinesis	Cytokinesis, chromosome congression and alignment
KIF15 (HKLP2)	Kinesin-12	Spindle/pole/midzone	Metaphase	Bipolar spindle formation in absence of KIF11
KIF18B	Kinesin-8	Spindle/pole	Interphase, Metaphase	Chromosome congression and alignment microtubule depolymerization
KIF20A (MKLP2, RabK6)	Kinesin-6	Spindle/midzone /midbody	Anaphase, Cytokinesis	Cytokinesis
KIF23 (MKLP1, KNSL5)	Kinesin-6	Spindle/midzone /midbody	Telophase, Cytokinesis	Cytokinesis
KIFC1 (HEST, KNSL2)	Kinesin-14	Spindle/pole	Prophase	Chromosome congression and alignment bipolar spindle formation

## Data Availability

Not applicable.

## Supplementary Materials

The following supporting information can be downloaded at: https://www.mdpi.com/article/10.3390/diagnostics12020470/s1.
