# Supplementary material for: Identifying Mitotic Kinesins as Potential Prognostic Biomarkers in Ovarian Cancer Using Bioinformatic Analyses
Source: Diagnostics (Basel). 2022 Feb 12;12(2):470. doi: 10.3390/diagnostics12020470 (PMC8871464; doi:10.3390/diagnostics12020470)
Supplement: Supplementary file 1 [file diagnostics-12-00470-s001.zip › diagnostics-1580768-supplementary.pdf]

Figure S1

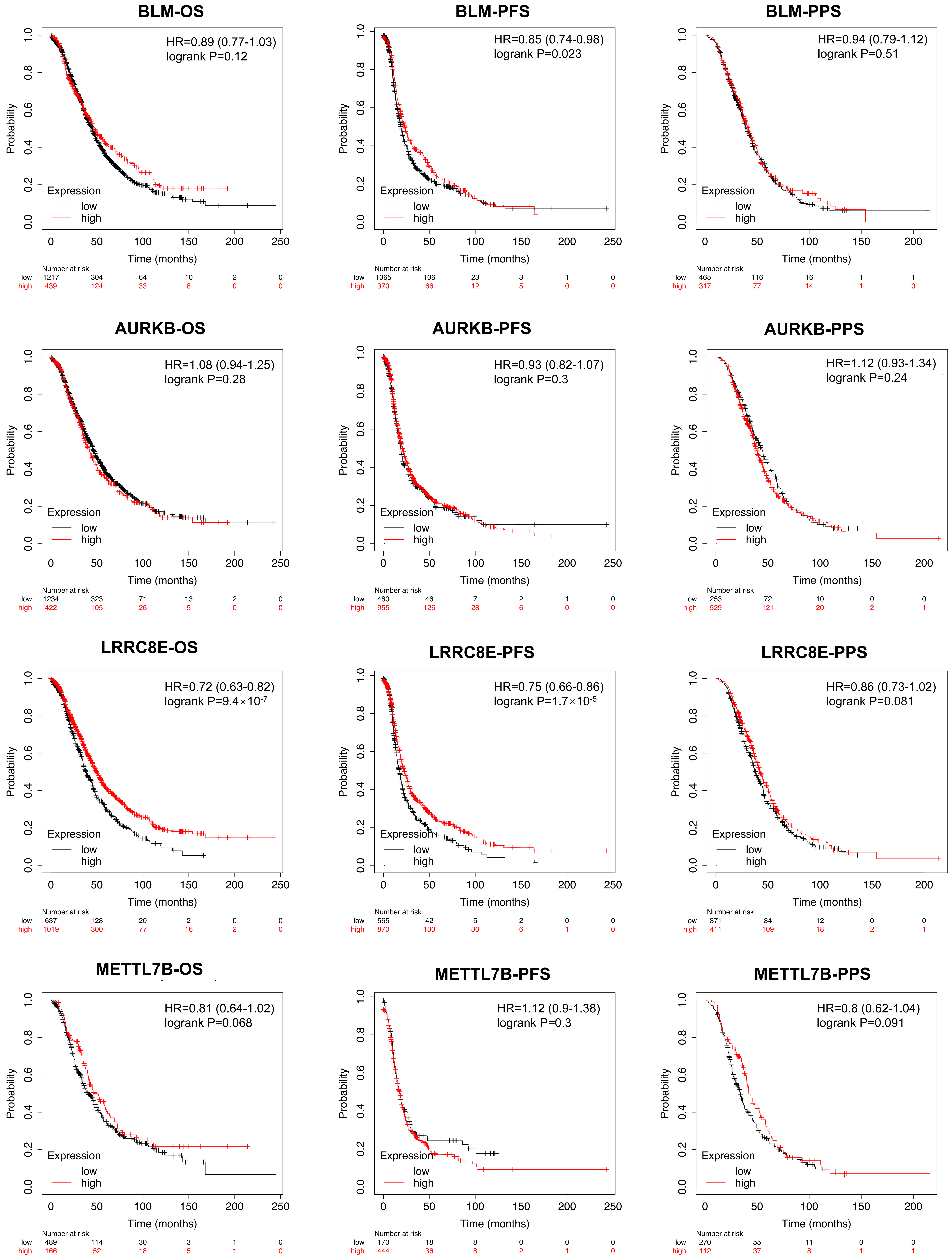
BLM-OS
HR=0.89 (0.77-1.03)
logrank P=0.12
Expression
low
high
Probability
Time (months)
Number at risk
low 1217 304 64 10 2 0
high 439 124 33 8 0 0
BLM-PFS
HR=0.85 (0.74-0.98)
logrank P=0.023
Number at risk
low 1065 106 23 3 1 0
high 370 66 12 5 0 0
BLM-PPS
HR=0.94 (0.79-1.12)
logrank P=0.51
Number at risk
low 465 116 16 1 1
high 317 77 14 1 0
AURKB-OS
HR=1.08 (0.94-1.25)
logrank P=0.28
Number at risk
low 1234 323 71 13 2 0
high 422 105 26 5 0 0
AURKB-PFS
HR=0.93 (0.82-1.07)
logrank P=0.3
Number at risk
low 480 46 7 2 1 0
high 955 126 28 6 0 0
AURKB-PPS
HR=1.12 (0.93-1.34)
logrank P=0.24
Number at risk
low 253 72 10 0 0
high 529 121 20 2 1
LRRC8E-OS
HR=0.72 (0.63-0.82)
logrank P=9.4×10-7
Number at risk
low 637 128 20 2 0 0
high 1019 300 77 16 2 0
LRRC8E-PFS
HR=0.75 (0.66-0.86)
logrank P=1.7×10-5
Number at risk
low 565 42 5 2 0 0
high 870 130 30 6 1 0
LRRC8E-PPS
HR=0.86 (0.73-1.02)
logrank P=0.081
Number at risk
low 371 84 12 0 0
high 411 109 18 2 1
METTL7B-OS
HR=0.81 (0.64-1.02)
logrank P=0.068
Number at risk
low 489 114 30 3 1 0
high 166 52 18 5 1 0
METTL7B-PFS
HR=1.12 (0.9-1.38)
logrank P=0.3
Number at risk
low 170 18 8 0 0 0
high 444 36 8 2 1 0
METTL7B-PPS
HR=0.8 (0.62-1.04)
logrank P=0.091
Number at risk
low 270 55 11 0 0
high 112 37 8 1 1

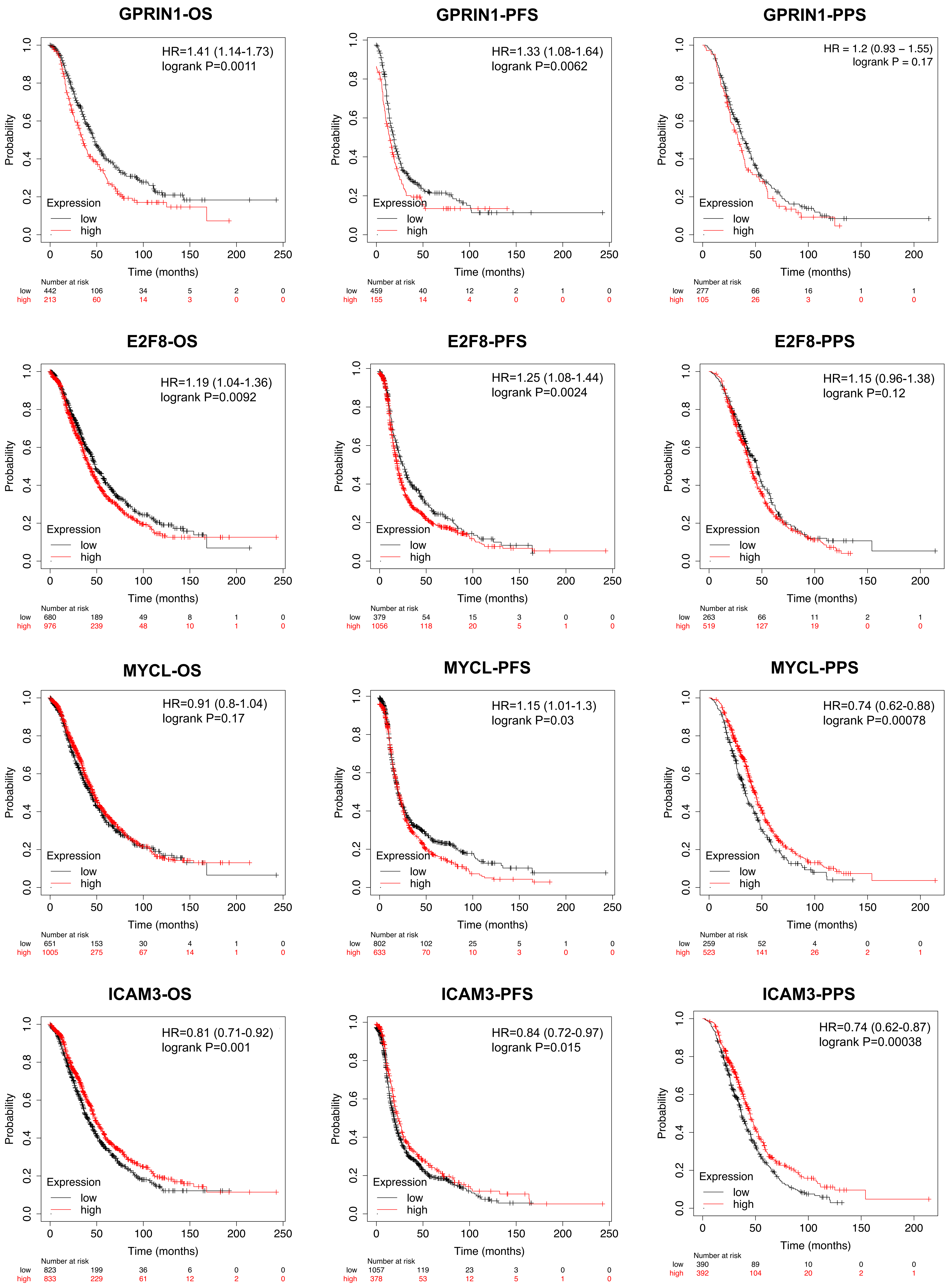
GPRIN1-OS
HR=1.41 (1.14-1.73)
logrank P=0.0011
Expression
low
high
Probability
Time (months)
Number at risk
low 442 106 34 5 2 0
high 213 60 14 3 0 0
GPRIN1-PFS
HR=1.33 (1.08-1.64)
logrank P=0.0062
Number at risk
low 459 40 12 2 1 0
high 155 14 4 0 0 0
GPRIN1-PPS
HR = 1.2 (0.93 − 1.55)
logrank P = 0.17
Number at risk
low 277 66 16 1 1
high 105 26 3 0 0
E2F8-OS
HR=1.19 (1.04-1.36)
logrank P=0.0092
Number at risk
low 680 189 49 8 1 0
high 976 239 48 10 1 0
E2F8-PFS
HR=1.25 (1.08-1.44)
logrank P=0.0024
Number at risk
low 379 54 15 3 0 0
high 1056 118 20 5 1 0
E2F8-PPS
HR=1.15 (0.96-1.38)
logrank P=0.12
Number at risk
low 263 66 11 2 1
high 519 127 19 0 0
MYCL-OS
HR=0.91 (0.8-1.04)
logrank P=0.17
Number at risk
low 651 153 30 4 1 0
high 1005 275 67 14 1 0
MYCL-PFS
HR=1.15 (1.01-1.3)
logrank P=0.03
Number at risk
low 802 102 25 5 1 0
high 633 70 10 3 0 0
MYCL-PPS
HR=0.74 (0.62-0.88)
logrank P=0.00078
Number at risk
low 259 52 4 0 0
high 523 141 26 2 1
ICAM3-OS
HR=0.81 (0.71-0.92)
logrank P=0.001
Number at risk
low 823 199 36 6 0 0
high 833 229 61 12 2 0
ICAM3-PFS
HR=0.84 (0.72-0.97)
logrank P=0.015
Number at risk
low 1057 119 23 3 0 0
high 378 53 12 5 1 0
ICAM3-PPS
HR=0.74 (0.62-0.87)
logrank P=0.00038
Number at risk
low 390 89 10 0 0
high 392 104 20 2 1

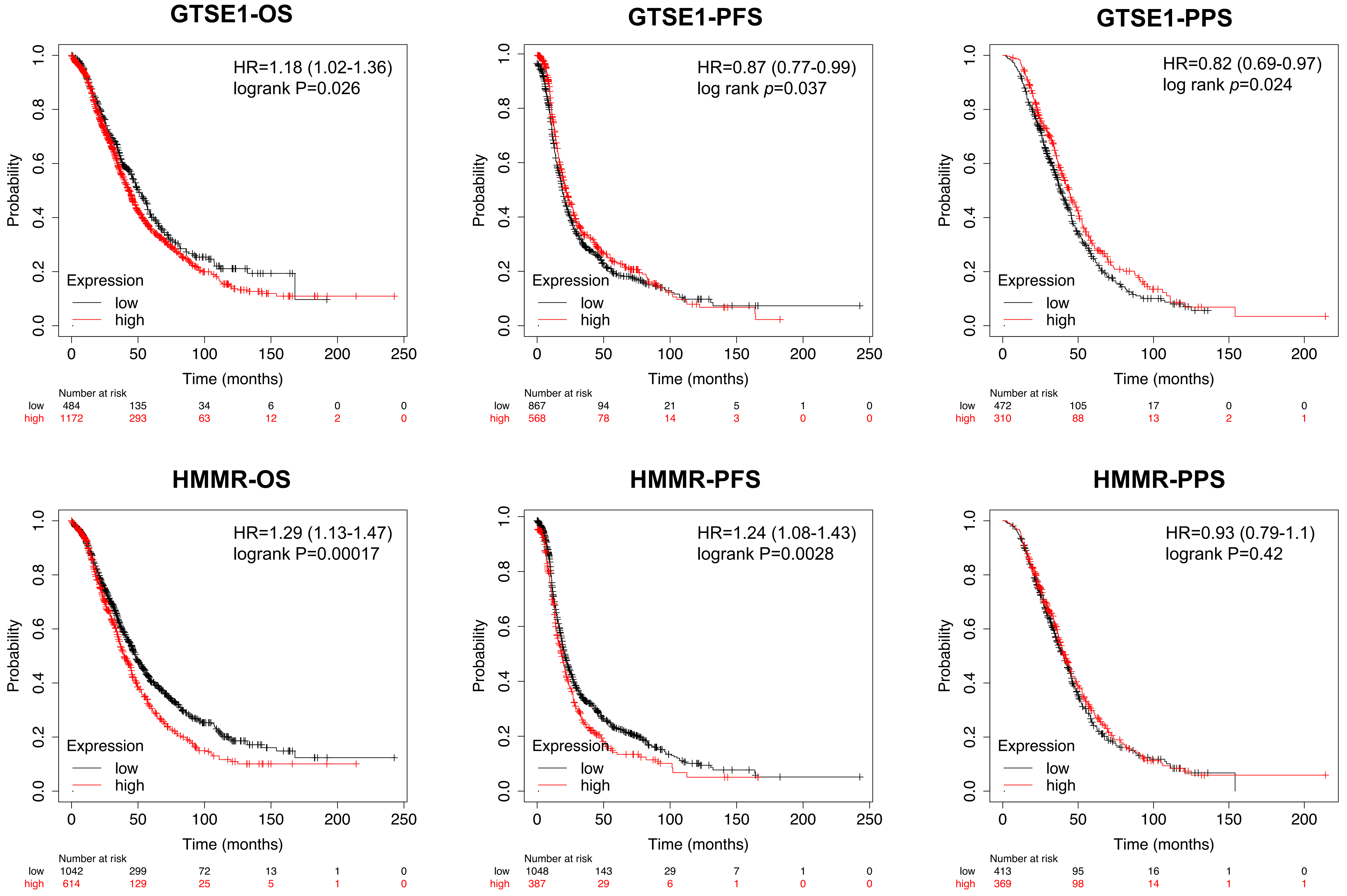

Figure S1.
Survival analysis of DEGs regarding OS (overall survival), PFS (post-free survival), and PPS (post-progression survival) using TCGA-OV dataset. Red, high-expression group; Black, low-expression group. *p*-value is log-ranked. Auto-selected best cutoff was used

Figure S2

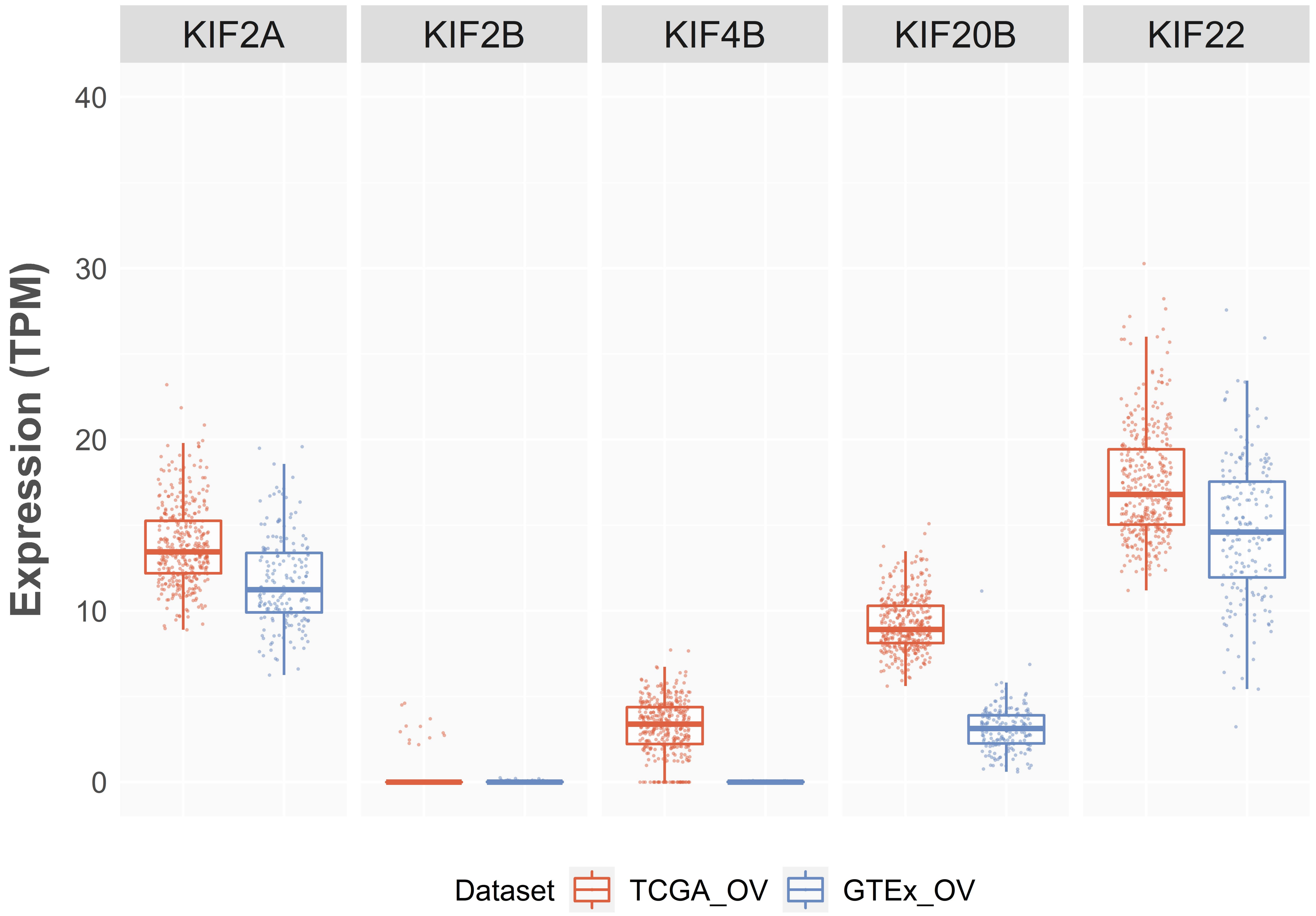

Figure S2.
Expression profiles of mitotic KIFs in OC.
No significant difference was obtained of mitotic KIFs (2A, 2B, 4B, 20B, and 22) between ovarian cancer and normal ovarian tissues from TCGA-OV (n=426) and GTEx-OV (n=88) dataset, respectively; TPM, transcripts per million; Expression of KIF10 was not detected in either ovarian cancer nor normal ovarian tissues.

Figure S3

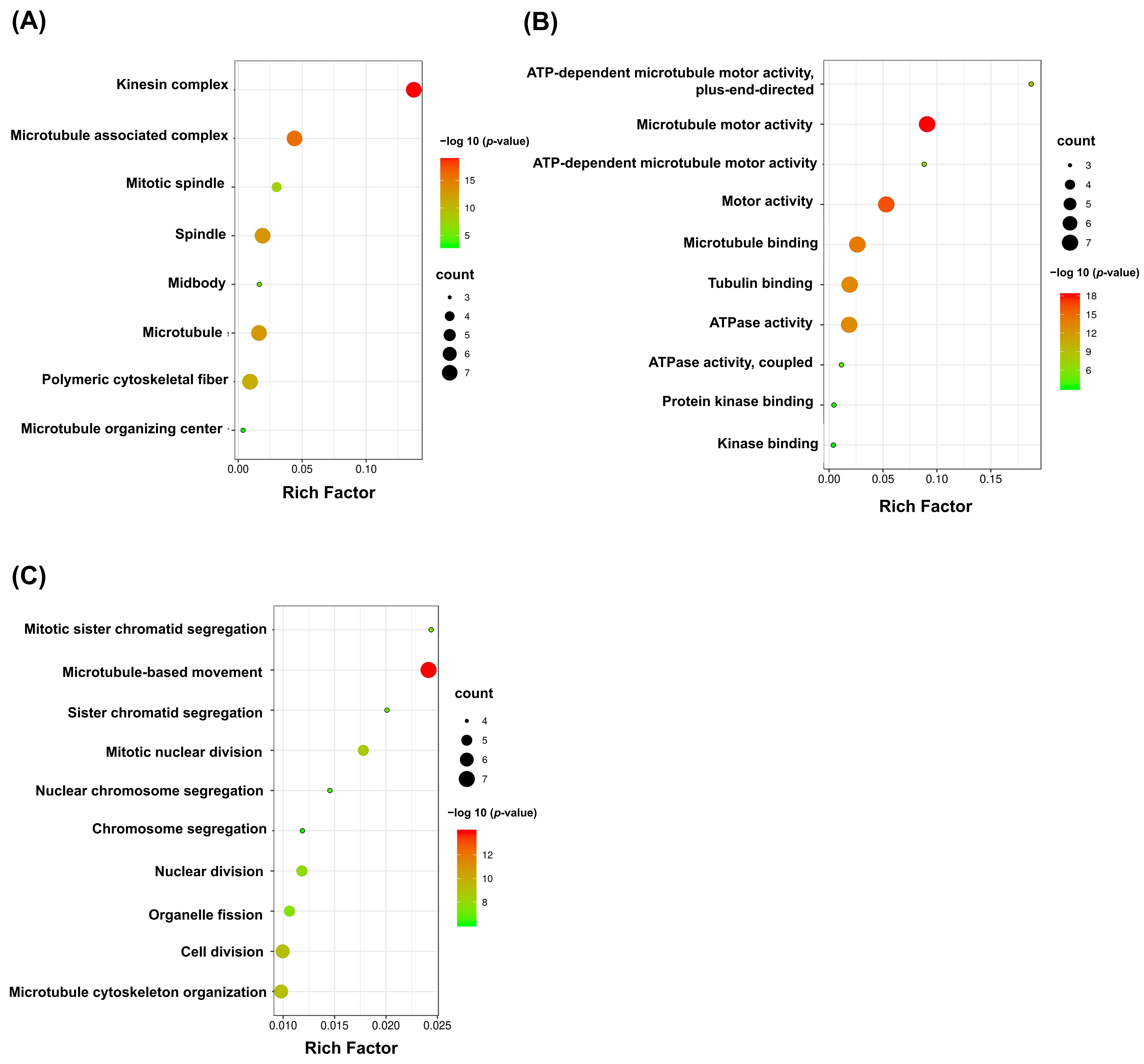

Figure S3. Top 10 GO functional enrichment analysis of overexpressed mitotic KIFs. (A) Cellular components; **(B)** Molecular function; **(C)** Biological pathways. Rich factor is the ratio of the DEGs number to the total gene numbers in a certain pathway. The color and size of the dots represent the range of *p*-value (hypergeometric test and Benjamini–Hochberg methods) and the number of DEGs mapped to the indicated results.

Figure S4

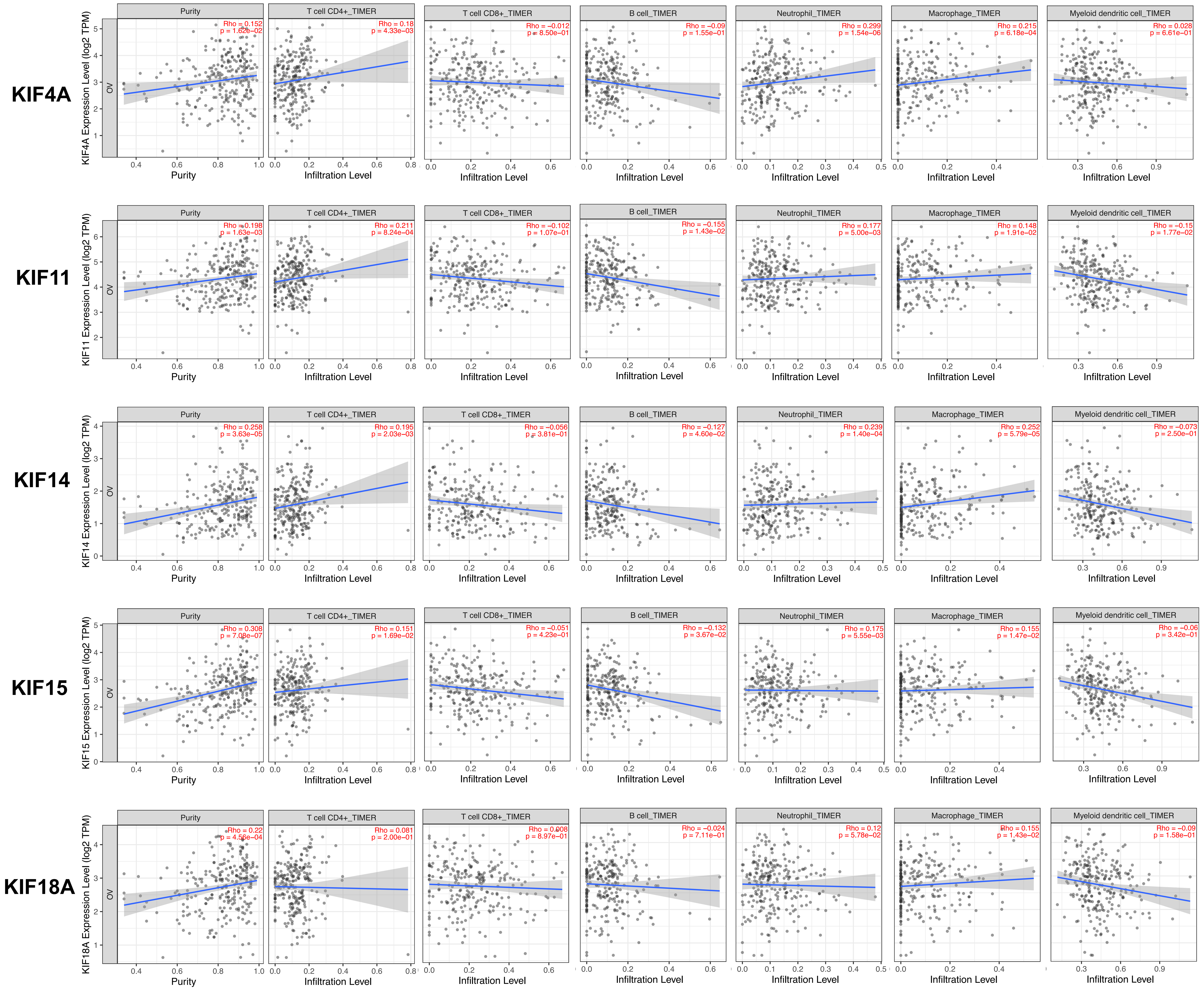

KIF4A
KIF11
KIF14
KIF15
KIF18A
OV
KIF4A Expression Level (log2 TPM)
KIF11 Expression Level (log2 TPM)
KIF14 Expression Level (log2 TPM)
KIF15 Expression Level (log2 TPM)
KIF18A Expression Level (log2 TPM)
Purity
T cell CD4+_TIMER
T cell CD8+_TIMER
B cell_TIMER
Neutrophil_TIMER
Macrophage_TIMER
Myeloid dendritic cell_TIMER
Infiltration Level
Rho = 0.152 p = 1.62e−02
Rho = 0.18 p = 4.33e−03
Rho = −0.012 p = 8.50e−01
Rho = −0.09 p = 1.55e−01
Rho = 0.299 p = 1.54e−06
Rho = 0.215 p = 6.18e−04
Rho = 0.028 p = 6.61e−01
Rho = 0.198 p = 1.63e−03
Rho = 0.211 p = 8.24e−04
Rho = −0.102 p = 1.07e−01
Rho = −0.155 p = 1.43e−02
Rho = 0.177 p = 5.00e−03
Rho = 0.148 p = 1.91e−02
Rho = −0.15 p = 1.77e−02
Rho = 0.258 p = 3.63e−05
Rho = 0.195 p = 2.03e−03
Rho = −0.056 p = 3.81e−01
Rho = −0.127 p = 4.60e−02
Rho = 0.239 p = 1.40e−04
Rho = 0.252 p = 5.79e−05
Rho = −0.073 p = 2.50e−01
Rho = 0.308 p = 7.08e−07
Rho = 0.151 p = 1.69e−02
Rho = −0.051 p = 4.23e−01
Rho = −0.132 p = 3.67e−02
Rho = 0.175 p = 5.55e−03
Rho = 0.155 p = 1.47e−02
Rho = −0.06 p = 3.42e−01
Rho = 0.22 p = 4.56e−04
Rho = 0.081 p = 2.00e−01
Rho = 0.008 p = 8.97e−01
Rho = −0.024 p = 7.11e−01
Rho = 0.12 p = 5.78e−02
Rho = 0.155 p = 1.43e−02
Rho = −0.09 p = 1.58e−01

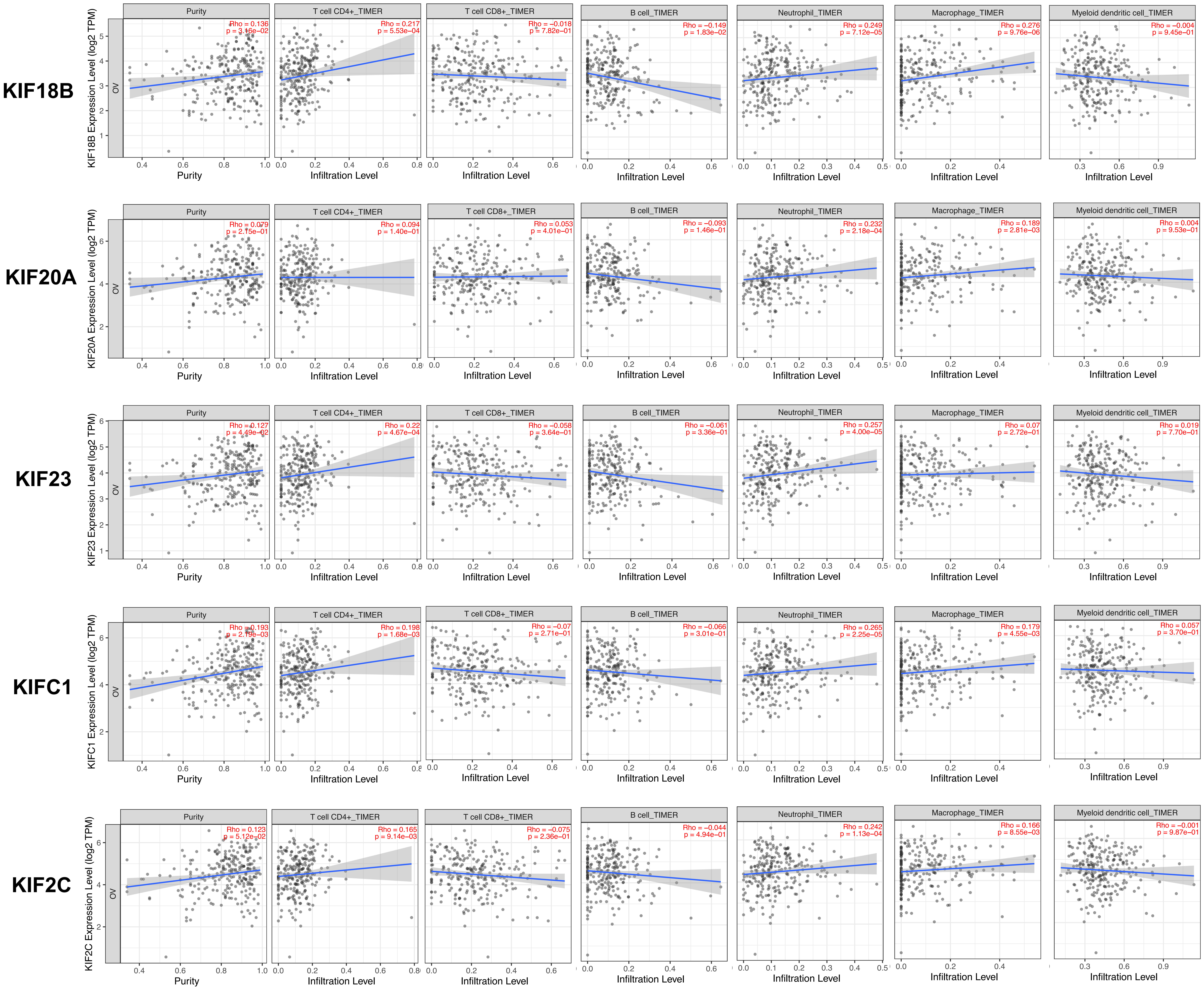

Figure S4. Correlation analysis between the overexpressed mitotic KIFs and common immune cells in OC using TIMER2.0 database.

Figure S5

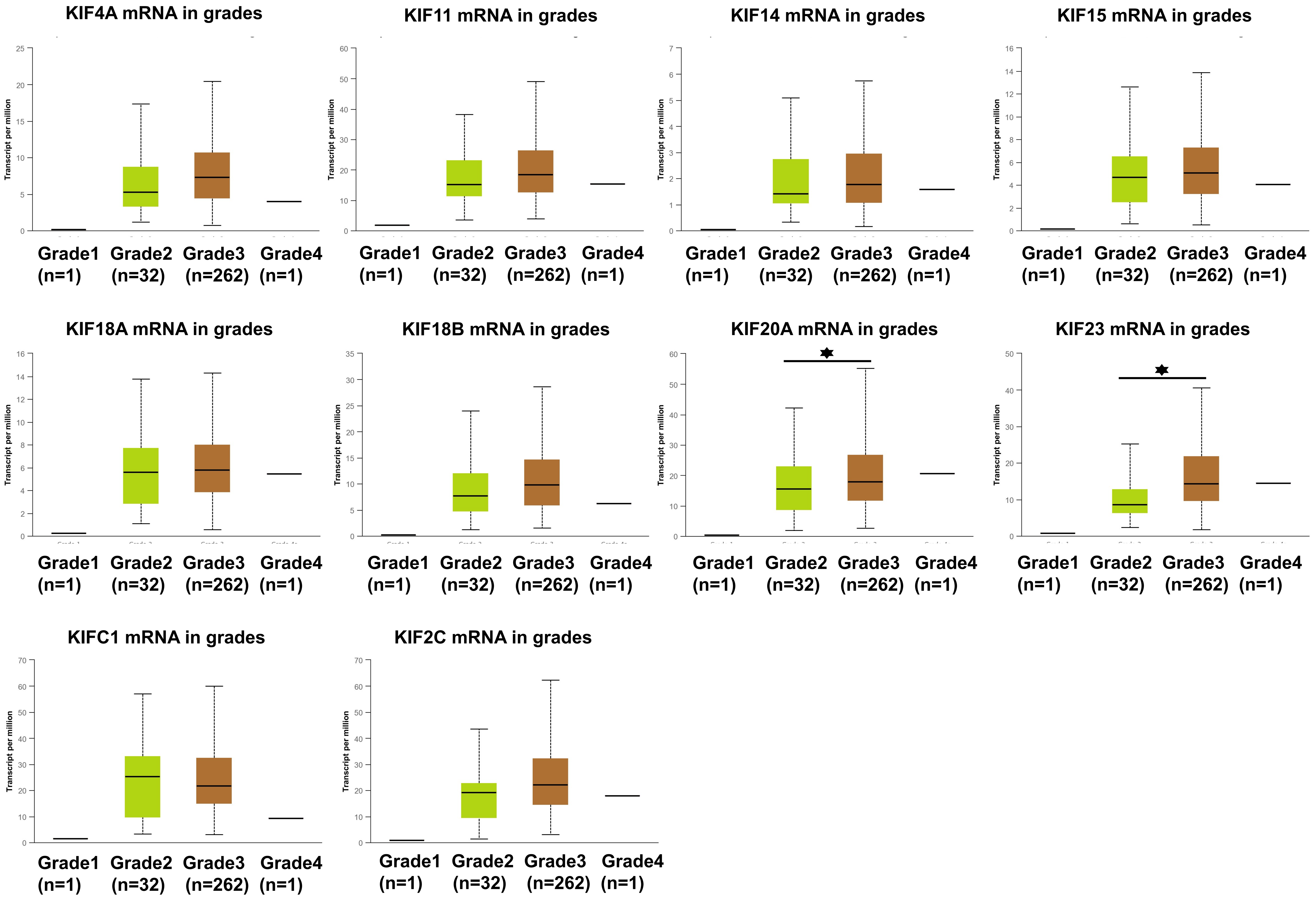

Figure S5. Transcription profiles of ten overexpressed mitotic KIFs in OC clinicopathological subgroup analysis (UALCAN). Sample numbers of each grade: grade 1(n=1); grade 2 (n=32); grade 3 (n=262); grade 4 (n=1). **p*<0.05.

Figure S6

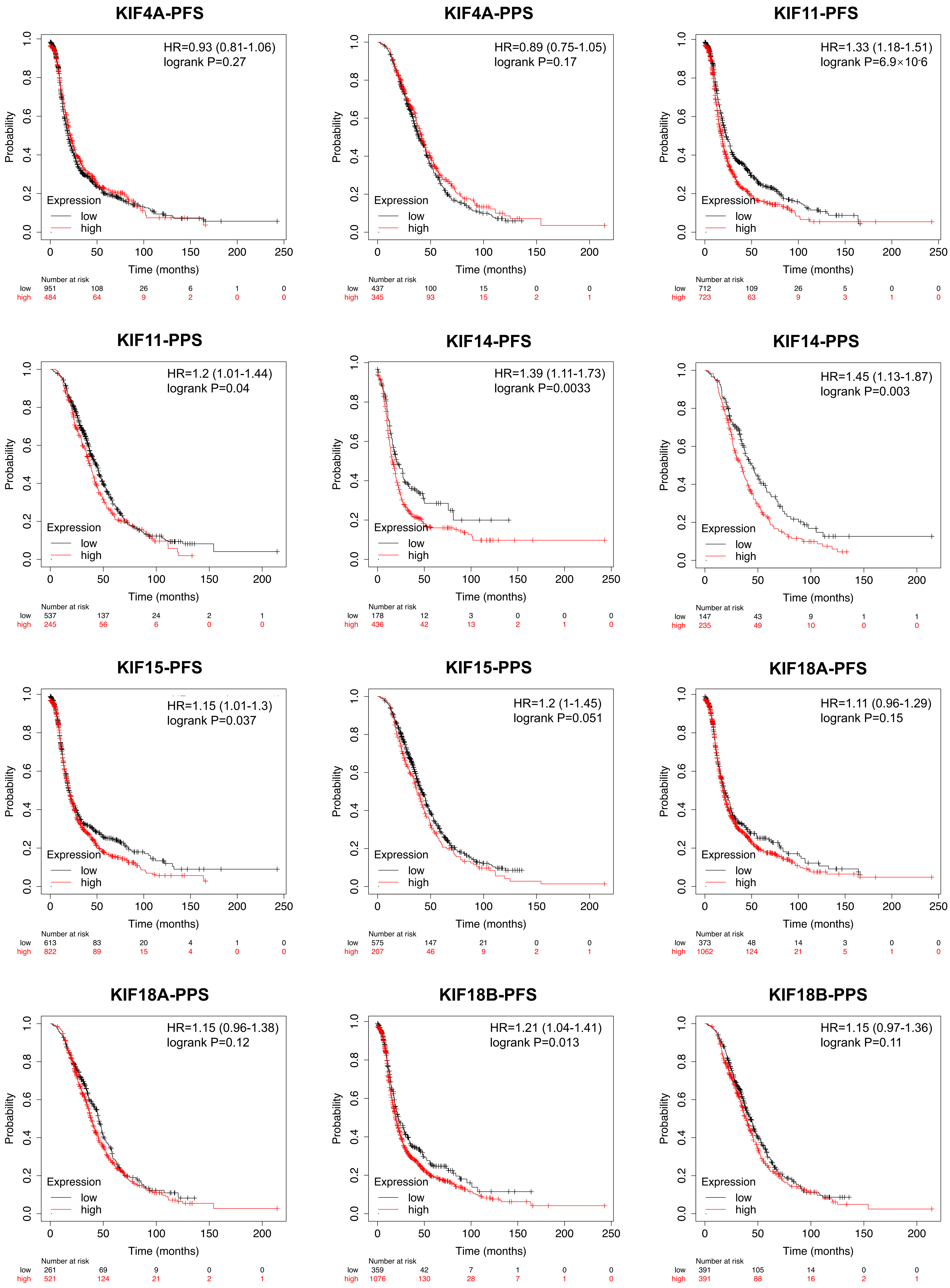
KIF4A-PFS
HR=0.93 (0.81-1.06)
logrank P=0.27
Expression
low
high
Probability
Time (months)
Number at risk
low 951 108 26 6 1 0
high 484 64 9 2 0 0
KIF4A-PPS
HR=0.89 (0.75-1.05)
logrank P=0.17
Expression
low
high
Probability
Time (months)
Number at risk
low 437 100 15 0 0
high 345 93 15 2 1
KIF11-PFS
HR=1.33 (1.18-1.51)
logrank P=6.9×10-6
Expression
low
high
Probability
Time (months)
Number at risk
low 712 109 26 5 0 0
high 723 63 9 3 1 0
KIF11-PPS
HR=1.2 (1.01-1.44)
logrank P=0.04
Expression
low
high
Probability
Time (months)
Number at risk
low 537 137 24 2 1
high 245 56 6 0 0
KIF14-PFS
HR=1.39 (1.11-1.73)
logrank P=0.0033
Expression
low
high
Probability
Time (months)
Number at risk
low 178 12 3 0 0 0
high 436 42 13 2 1 0
KIF14-PPS
HR=1.45 (1.13-1.87)
logrank P=0.003
Expression
low
high
Probability
Time (months)
Number at risk
low 147 43 9 1 1
high 235 49 10 0 0
KIF15-PFS
HR=1.15 (1.01-1.3)
logrank P=0.037
Expression
low
high
Probability
Time (months)
Number at risk
low 613 83 20 4 1 0
high 822 89 15 4 0 0
KIF15-PPS
HR=1.2 (1-1.45)
logrank P=0.051
Expression
low
high
Probability
Time (months)
Number at risk
low 575 147 21 0 0
high 207 46 9 2 1
KIF18A-PFS
HR=1.11 (0.96-1.29)
logrank P=0.15
Expression
low
high
Probability
Time (months)
Number at risk
low 373 48 14 3 0 0
high 1062 124 21 5 1 0
KIF18A-PPS
HR=1.15 (0.96-1.38)
logrank P=0.12
Expression
low
high
Probability
Time (months)
Number at risk
low 261 69 9 0 0
high 521 124 21 2 1
KIF18B-PFS
HR=1.21 (1.04-1.41)
logrank P=0.013
Expression
low
high
Probability
Time (months)
Number at risk
low 359 42 7 1 0 0
high 1076 130 28 7 1 0
KIF18B-PPS
HR=1.15 (0.97-1.36)
logrank P=0.11
Expression
low
high
Probability
Time (months)
Number at risk
low 391 105 14 0 0
high 391 88 16 2 1

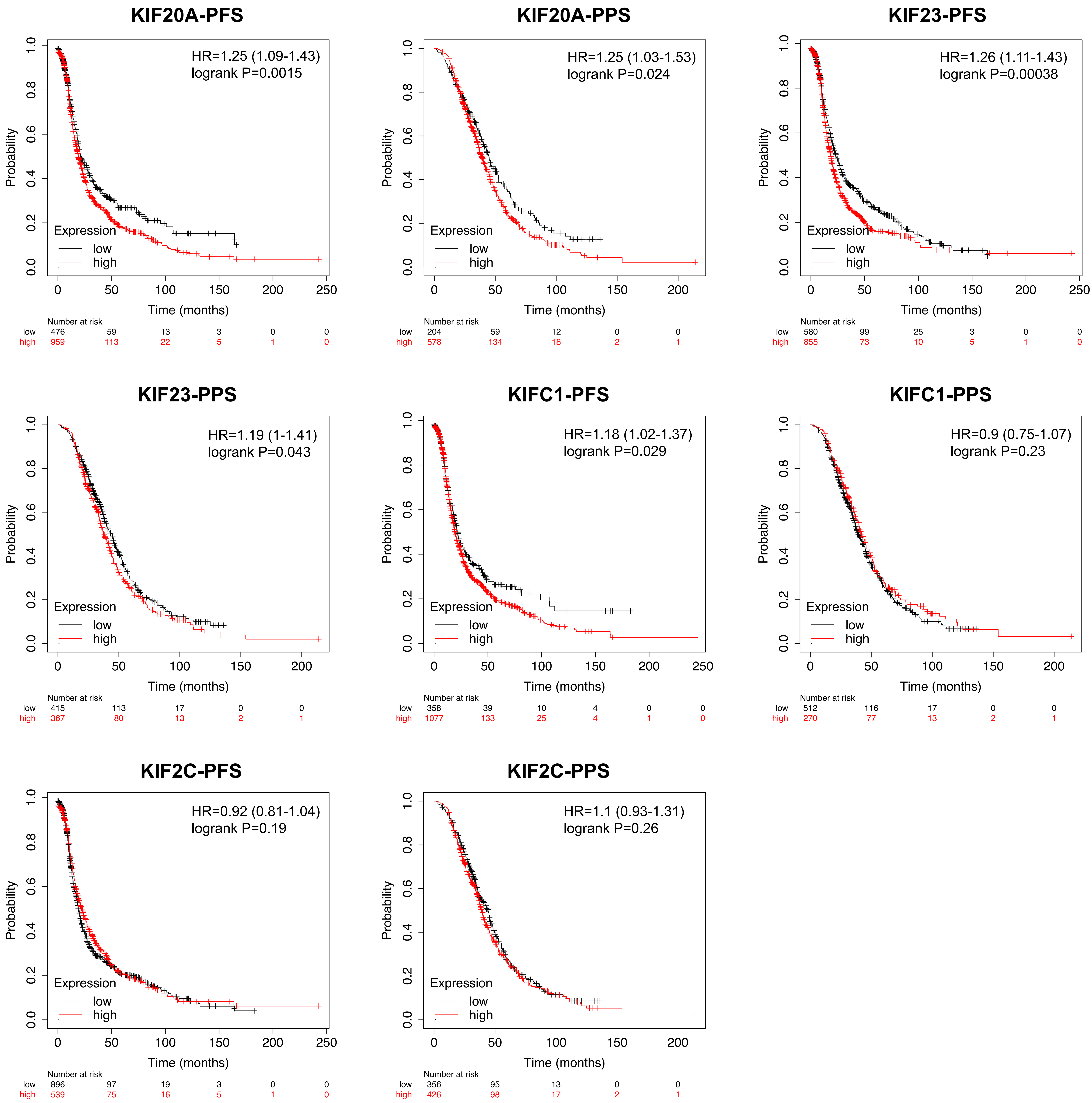

Figure S6.
Survival analysis of mitotic KIFs regarding PFS (post-free survival) and PPS (post-progression survival) using TCGA-OV dataset. Red, high-expression group; Black, low-expression group. *p*-value is log-ranked. Auto-selected best cutoff was used
